# A Simple, Single-Staged Pedicled Rotational Flap With Lateral Band Tendon Graft for the Reconstruction of Composite Dorsal Finger Defects

**DOI:** 10.7759/cureus.97978

**Published:** 2025-11-27

**Authors:** Ying Beatrice Lim, Min Kai Chang, Fang Li

**Affiliations:** 1 Department of Plastic, Reconstructive and Aesthetic Surgery, Singapore General Hospital, Singapore, SGP; 2 Department of Hand and Reconstructive Microsurgery, Singapore General Hospital, Singapore, SGP

**Keywords:** composite, dorsal, extensor, finger, flap, hand surgery, orthopedic, plastic surgery, reconstruction, tendon

## Abstract

Composite defects over the dorsal finger are challenging to reconstruct, especially when they involve the eponychium and extensor tendon. We present a case series involving three patients where a single-stage pedicled rotational flap with lateral band tendon graft was used for the reconstruction of the composite defect at the dorsal distal or middle phalanx of the fingers. The flap was based on the dorsal skin branches of the digital arteries at the proximal phalanx and can extend distally to cover eponychial defects. Two of the three patients also had an extensor apparatus defect of 1.0 cm or more and were successfully reconstructed with a lateral band tendon graft with good functional outcomes. The flaps healed uneventfully. This flap with tendon graft technique offers a straightforward, reliable, single-stage reconstruction, providing like-for-like tissue replacement with minimal donor site morbidity.

## Introduction

Reconstruction of dorsal finger defects poses a unique challenge. Unlike the volar skin, the dorsal finger skin is thin with a scarcity of available tissue. Owing to thin overlying skin, traumatic defects in this region are frequently composite defects involving the skin, extensor tendon, and/or bone. Compared to the various options for volar finger defects, the limited soft tissue on the dorsal finger makes it challenging to recruit adjacent tissue for primary closure or local advancement flaps. Commonly described techniques include the reverse dorsal metacarpal artery flap [1] and the reverse cross-finger flap [2]. However, most current reconstructive approaches have one or more notable drawbacks: (1) compromising an uninjured area, such as an adjacent finger; (2) requiring staged procedures to divide pedicled flaps; (3) necessitating skin grafts to cover donor sites; and (4) involving complex microsurgical procedures, such as free flaps.

Composite defects involving the extensor tendon in zones 1 and 2 often result in extensor lag that impairs function and leads to mallet finger and swan neck deformities. Even a 1-mm change in the length of the extensor mechanism can seriously affect finger joint motion [3]. Therefore, precise restoration of the extensor mechanism is important for finger function. Terminal extensor tendon reconstruction techniques include tendon grafts [4] and extensor retinaculum grafts [5], which result in donor site morbidity. Techniques using tendon flaps from lateral bands have also been described, but they are mostly limited to small extensor tendon defects of less than 1.0 cm [6-8].

In this case series, we describe a simple, single-stage rotational flap based on the dorsal branch of the digital artery of the proximal phalanx, combined with a lateral band tendon graft for coverage of composite distal dorsal finger defects with a large extensor tendon gap of more than 1.0 cm. The flap avoids the aforementioned disadvantages and can also be used to reconstruct the eponychium.

## Case presentation

Surgical technique

Pedicled Rotational Flap

The indication for this technique is a dorsal distal phalanx and/or middle phalanx defect with or without extensor tendon defect, exposed joint capsule, or phalanx bone. For distal dorsal finger defects, an eponychial defect is also an indication. The procedure is performed under regional anesthesia and tourniquet control. The rotational flap is elevated from the dorsoulnar or dorsoradial aspect of the affected finger in a subfascial plane. The paratenon is left intact. A curvilinear incision is made, beginning at the distal edge of the defect and extending along the midlateral line. The distal edge of the flap can be as distal as the eponychium, as shown in one of our cases for eponychial reconstruction. As the incision nears the proximal half of the proximal phalanx, it is curved back over the dorsum, avoiding the interdigital webspace, and ends near the midline of the metacarpophalangeal joint. The dorsal skin branch of the proper digital artery at the level of the proximal phalanx shaft is carefully preserved.

The lateral margin of the flap can also be extended past the midlateral line to recruit more volar skin if necessary, taking care not to jeopardize the proper digital neurovascular bundle. The flap is elevated in the plane superficial to the extensor tendon to preserve the subdermal vascular plexus. Care is taken when dissecting the base of the flap to avoid jeopardizing the dorsal branches of the digital artery. The flap is rotated to cover the dorsal defect and is inset with loose sutures to minimize tension.

Lateral Band Tendon Graft

**Figure 1:**
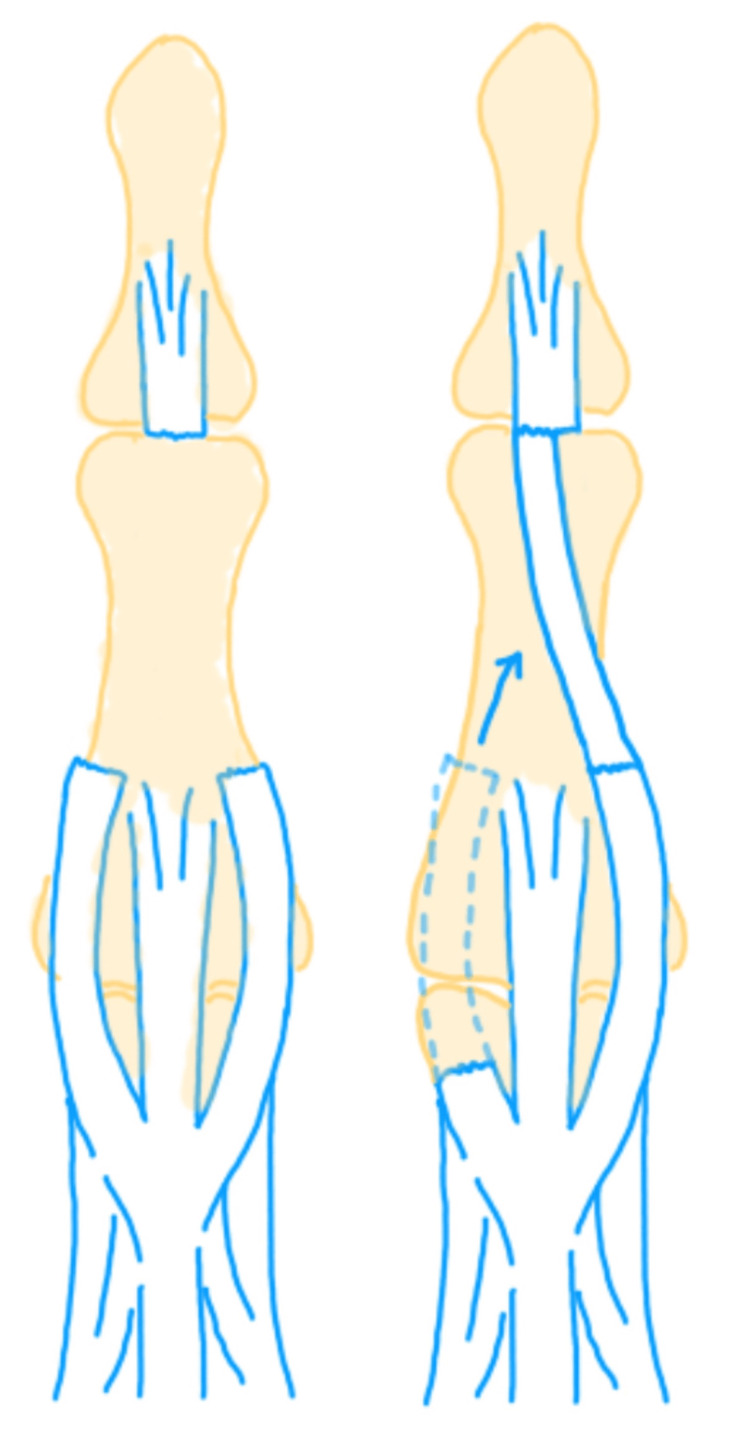
Extensor tendon reconstruction using lateral band tendon graft This figure was created by the authors

After the pedicled rotation skin flap is raised, the extensor apparatus with its defect is exposed. The ends of the tendon are debrided. The radial lateral band and ulnar lateral band defects are measured accurately. The proximal radial or ulnar lateral band is harvested as a tendon graft to bridge the gap. If the radial lateral band is harvested, it is used to suture the proximal ulnar lateral band to the distal radial lateral band insertion, and vice versa (Figure 1). The distal interphalangeal joint (DIPJ) is temporarily fixed with a Kirschner wire in extension before the reconstruction. This wire is removed after four weeks. After K-wire removal, the finger is splinted for another two weeks, with intermittent removal for active mobilization.

Case illustrations

Patient 1

**Figure 2:**
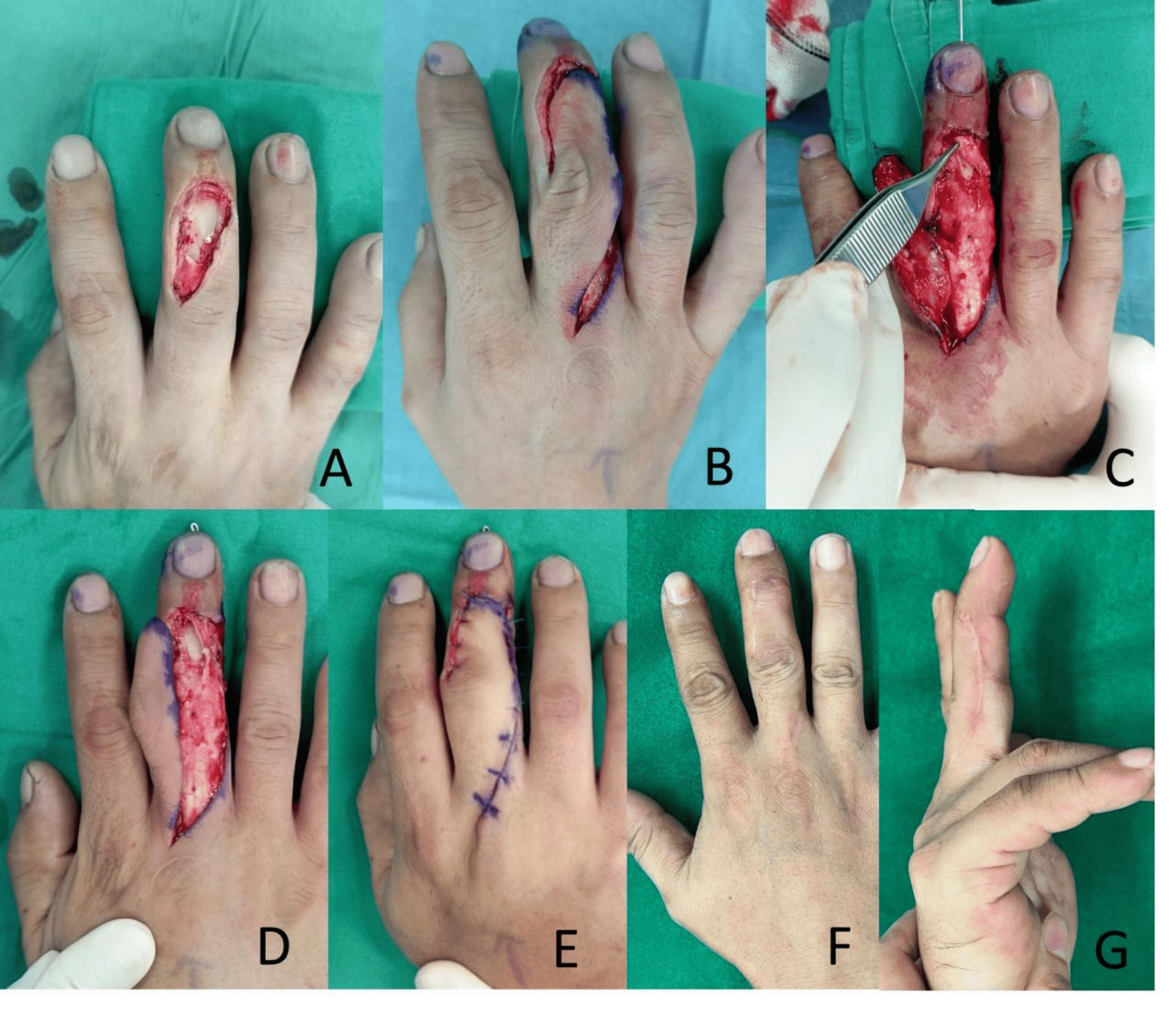
Patient 1 A: The 3.0 x 1.5 cm dorsal defect after debridement. B: After elevation of the rotation flap. C, D: Repair of the extensor tendon using the radial lateral band. E: After the insertion of the flap. F: Postoperative appearance at six months. G: Finger in extension

A 38-year-old male sustained a right middle finger shaving injury after a fall, resulting in a dorsal defect of the middle phalanx exposing the bone and the DIPJ (Figure 2). He was unable to extend the DIPJ. Intraoperatively, both lateral bands of the extensor tendon were cut. The radial lateral band defect was 2.0 cm, and the ulnar lateral band defect was 1.2 cm. The central slip was intact. The final soft tissue defect after debridement was 3.0 x 1.5 cm over the dorsoradial finger. The surgery was performed on the same day as the injury. The radial lateral band was harvested as a tendon graft and used to bridge the gap between the proximal ulnar lateral band and the distal radial lateral band insertion. A rotation flap was elevated from the dorsoulnar aspect of the finger, which healed fully. After six months, the patient had a DIPJ extension lag of 20 degrees and full flexion of 70 degrees. He was satisfied with the results and returned to his regular duties at work as an aircraft engineer. No postoperative complications were noted.

Patient 2

**Figure 3:**
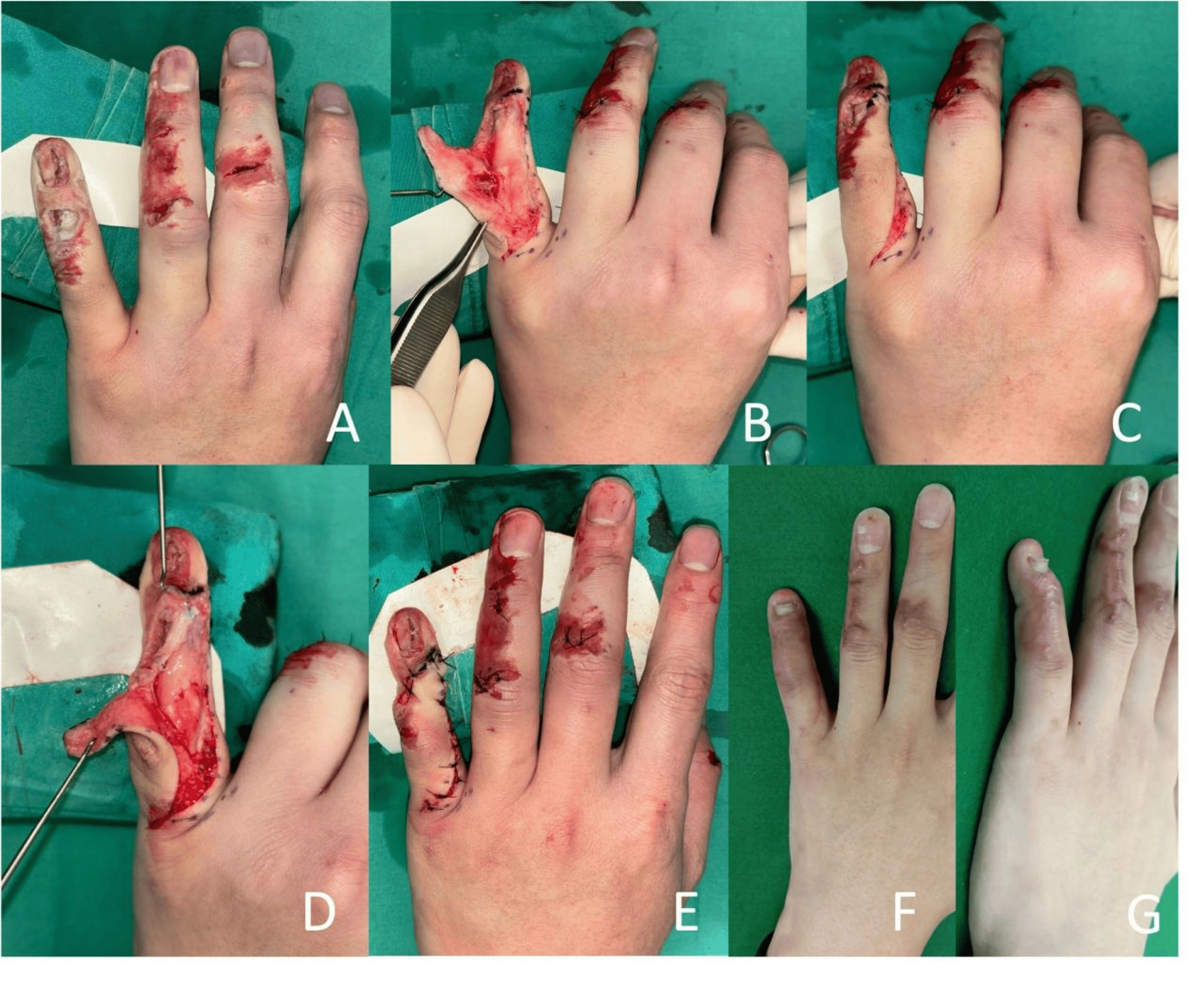
Patient 2 A: The 1.0 x 1.0 cm dorsal defect after debridement. B, C: After the elevation of the rotation flap. D: After the extensor tendon repair. E: After the insertion of the flap. F: Postoperative appearance at four months. G: Finger in extension

A 37-year-old male sustained a left little finger injury from a cycling accident, resulting in a dorsal defect exposing the DIPJ (Figure 3). He was unable to extend the DIPJ. Radiographs showed a tuft fracture, anterior subluxation of the distal phalanx, and a minimally displaced fracture of the middle phalanx ulnar head. Intraoperatively, lateral bands of the extensor tendon were disrupted in zone 1 with a 1.0 cm defect. There was a 30% sterile matrix loss as well. The final skin defect after debridement was 1.0 x 1.0 cm, centred over the DIPJ. The extensor tendon was reconstructed in a similar fashion to Patient 1. The surgery was performed on the same day as the injury. A single axial K-wire was used to pin the DIPJ and fracture. The edges of the nailbed defect were approximated and closed. The soft tissue defect was covered with the rotation flap elevated from the dorsoradial finger. The flap healed fully. At four months, the patient demonstrated a 20-degree extension lag at the DIPJ with full flexion to 60 degrees. He was satisfied with the outcome, had resumed weight training, and reported no weakness in grip strength. No postoperative complications were reported.

Patient 3

**Figure 4:**
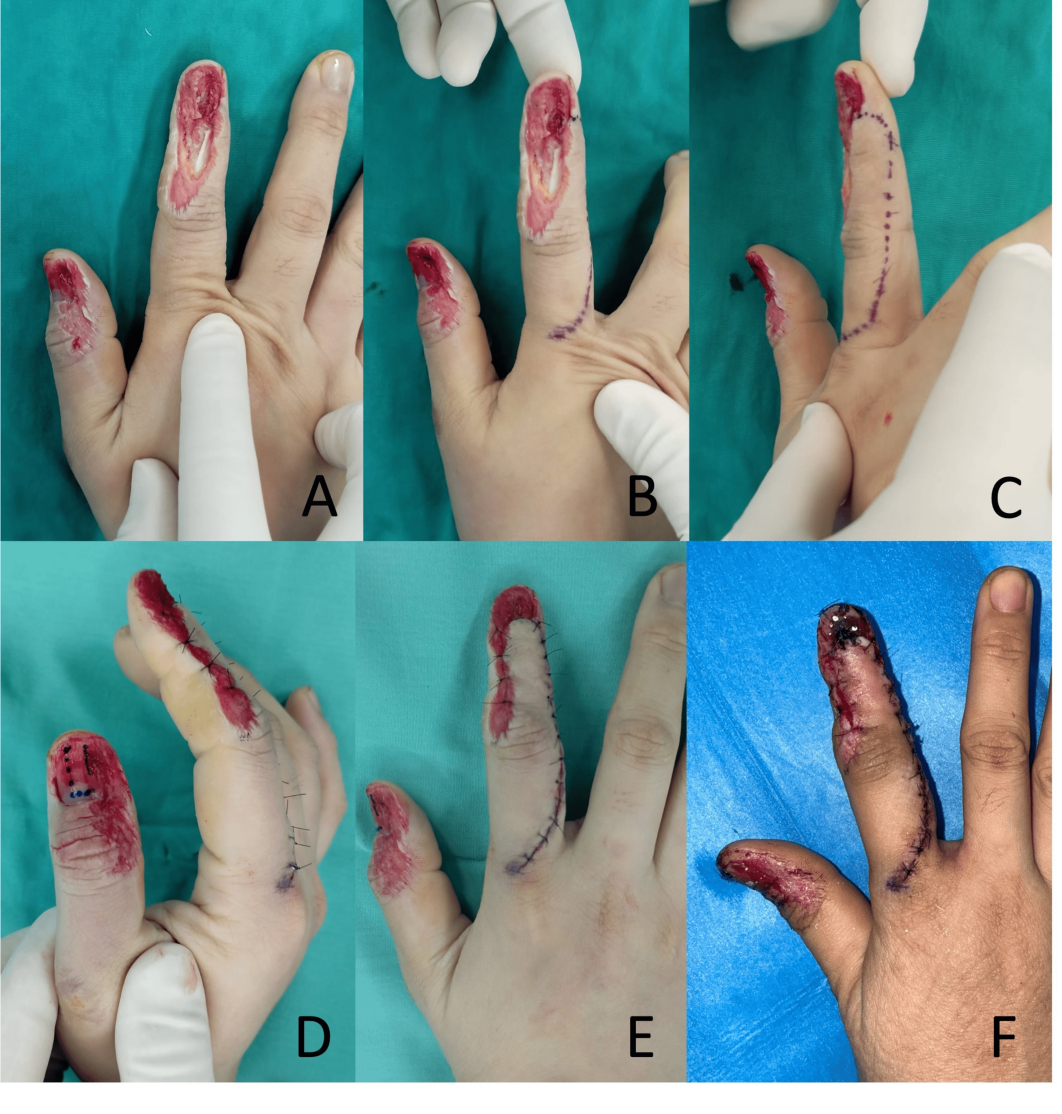
Patient 3 A: The 2.5 x 1.0 cm dorsal defect after debridement. B, C: Design of the rotation flap. D: Harvesting of the right thumb nailbed graft. E: After the flap insertion. F: Postoperative appearance

A 26-year-old female sustained a right index finger crush injury from a coconut press machine, resulting in a dorsal defect of the middle and distal phalanges, exposing the distal phalanx bone and DIPJ (Figure 4). There was a 1.0 cm eponychial defect and a sterile matrix defect measuring 1.2 x 0.7 cm. The radial lateral band of the extensor tendon was lost, but the ulnar lateral band was found to be intact. Hence, the extensor tendon was not reconstructed for this case. The soft tissue defect was predominantly over the radial dorsum, extending from the mid-middle phalanx to the nailbed, measuring 2.5 x 1.0 cm after debridement. The defect was covered with the rotation flap elevated from the dorsoulnar finger one day after the injury. The distal edge of the flap was used to recreate the eponychial fold. The nailbed defect was repaired with a nailbed graft harvested from the right thumb. The flap healed fully with a good aesthetic result of the eponychial fold and nail bed. No postoperative complications occurred.

## Discussion

Given the conspicuous location and the high demand for mobility, the ideal reconstruction method for dorsal finger defects should provide durable soft tissue coverage for tendon gliding, while minimizing bulkiness and scarring. Both the reverse dorsal metacarpal artery flap and the reverse cross-finger flap are viable options. The reverse cross-finger flap is robust and relatively simple to execute, but requires two stages and a skin graft for donor site coverage. It cannot be used for dorsal defects over the radial index finger or the ulnar little finger. The reverse dorsal metacarpal artery flap offers a single-stage option without the need for skin grafting. However, it may not reach distal defects, and distal tip necrosis secondary to venous congestion from kinking of the pedicle is a known complication.

This rotation flap addresses the above challenges by providing a single-stage option that is simple to execute, even for less-experienced surgeons. It requires neither tedious dissection of a vascular pedicle nor microsurgical skills or equipment. The donor site is closed primarily without skin grafting, leaving a surgical scar confined solely to the injured digit, with no additional donor site morbidity. The flap can also be used for eponychial reconstruction, which has few locoregional options [9]. The flap is reliable as it is based on the dorsal skin branch of the palmar digital artery (PDA) at the proximal phalanx [10]. In all three patients, the rotation flaps healed completely, with no compromise to distal vascularity despite a high length-to-width ratio. Our case series shows that as long as this branch is carefully preserved, a long flap can be reliably raised to cover distal defects even up to the eponychium. This is unlike a random pattern flap - where the recommended length-to-width ratio is 1:1 [11] and not exceeding 2:1 [12].

Large extensor tendon defects are challenging to reconstruct. To our knowledge, only one other published technique has successfully used the lateral band as a graft to bridge extensor tendon defects greater than 1.0 cm [7]. The described technique involved using half of each lateral band to bridge extensor tendon gaps of up to 2.0 cm. The reported final arc of motion of this technique was 37 degrees. All these techniques that used lateral bands as grafts reported a slight DIPJ extension lag as well [6-8].

Our technique of using the lateral band as a graft is simple and does not involve additional donor site morbidity. The lateral band graft crosses the midline to avoid lateral band subluxation. While there is a 20-degree extension lag of the DIPJ in our cases, the patients have reported a satisfactory functional arc of motion of 40 to 50 degrees. All patients were pleased with their outcomes and were able to return to their previous occupations.

## Conclusions

Dorsal finger defects with large extensor tendon gaps are challenging to reconstruct. The pedicled rotational flap, combined with a lateral band graft, offers aesthetic and functional coverage of the dorsal distal phalanx and middle phalanx soft tissue defects. This approach is a simple, reliable, single-stage procedure that avoids additional donor-site morbidity.
